# Correction: Suppression of choroidal neovascularization and epithelial-mesenchymal transition in retinal pigmented epithelium by adeno-associated virus-mediated overexpression of CCN5 in mice

**DOI:** 10.1371/journal.pone.0293237

**Published:** 2023-10-17

**Authors:** Sora Im, Jung Woo Han, Euy Jun Park, Ji Hong Bang, Hee Jeong Shin, Hun Soo Chang, Kee Min Woo, Woo Jin Park, Tae Kwann Park

There are errors in the Funding section. The correct Funding statement is: K.M.W was supported by grant from Business for Startup growth and technological development (TIPS Program, S2842182) funded by Korea Ministry of SMEs and Startups in 2020. W.J.P was supported by grant from the National Research Foundation of Korea (2022R1A4A2000767, 2022R1A2C1004256) funded by the Korean government (MSIT). MSIT is the abbreviation of Ministry of Science and ICT. The funder had no role in study design, data collection and analysis, decision to publish, or preparation of the manuscript.
