# Supplementary figures and images for: Suppression of choroidal neovascularization and epithelial-mesenchymal transition in retinal pigmented epithelium by adeno-associated virus-mediated overexpression of CCN5 in mice
Source: PLoS One. 2022 Jun 13;17(6):e0269937. doi: 10.1371/journal.pone.0269937 (PMC9191714; doi:10.1371/journal.pone.0269937)

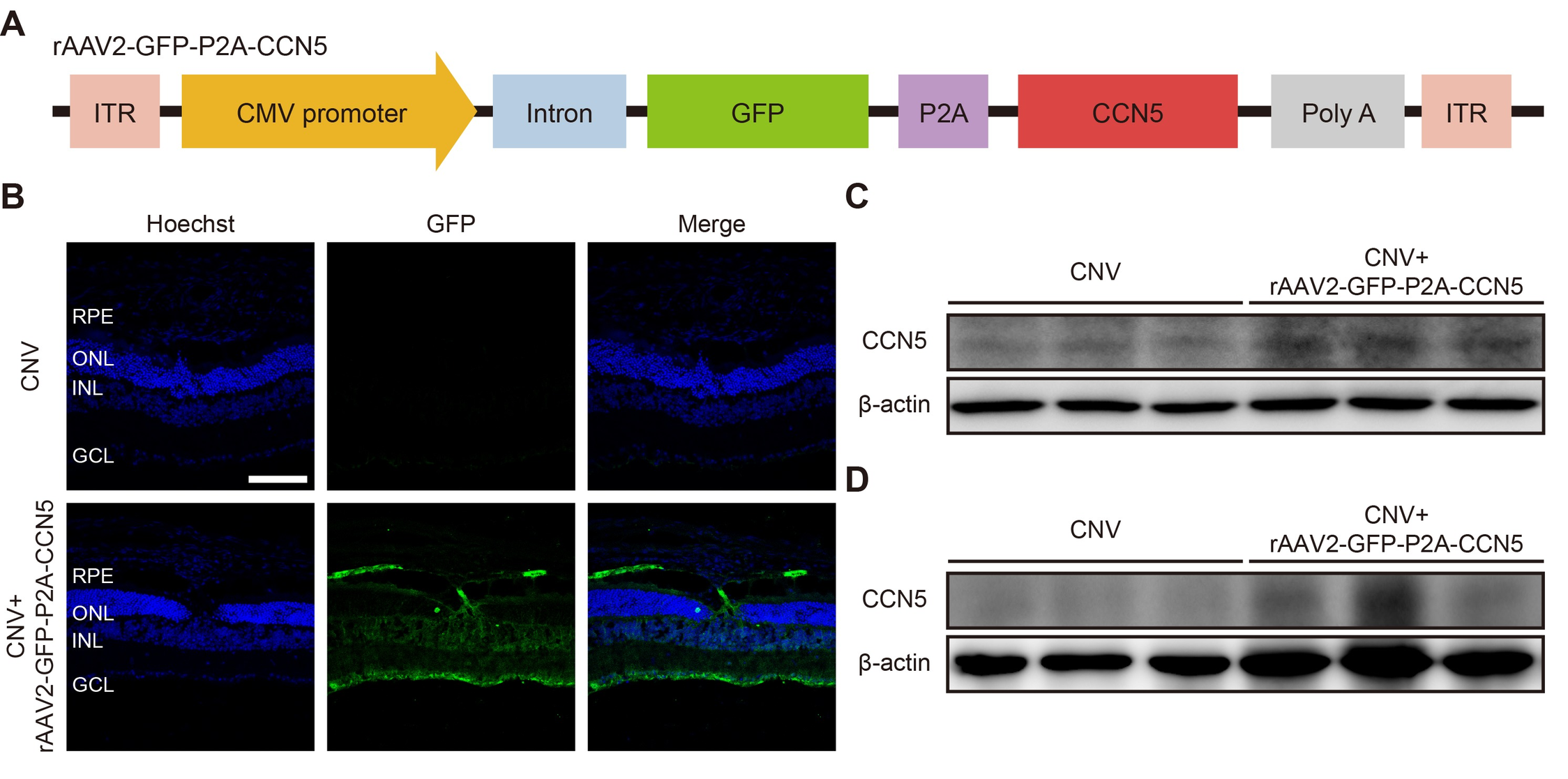

Supplement: S1 Fig — (A). A schematic representation of rAAV2-GFP-P2A-CCN5. (B). Immunostaining of GFP in transverse retinal sections. GFP expression was detectable from ganglion cell to RPE layer. Scale bar, 100 μm. (C, D). CCN5 expression was determined via western blotting from retina and RPE/choroid complex. (n = 3~4 mice per group). (TIF) [file pone.0269937.s001.tif]

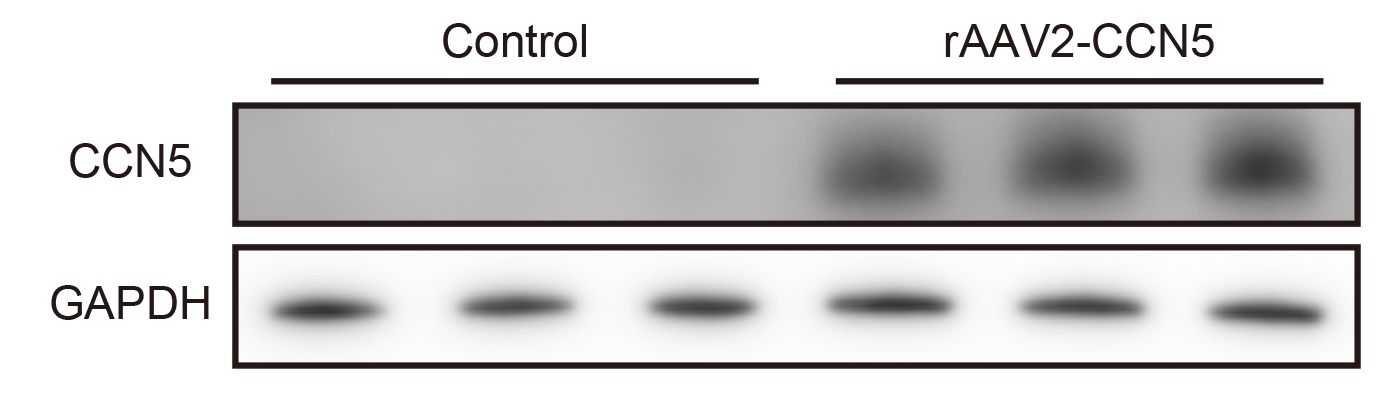

Supplement: S2 Fig — ARPE-19 cells were infected with rAAV2-CCN5 at MOI of 1,000 for 5 days. CCN5 expression was determined via western blotting from whole cells lysates. (n = 4 per group). (TIF) [file pone.0269937.s002.tif]

Figure 1-B

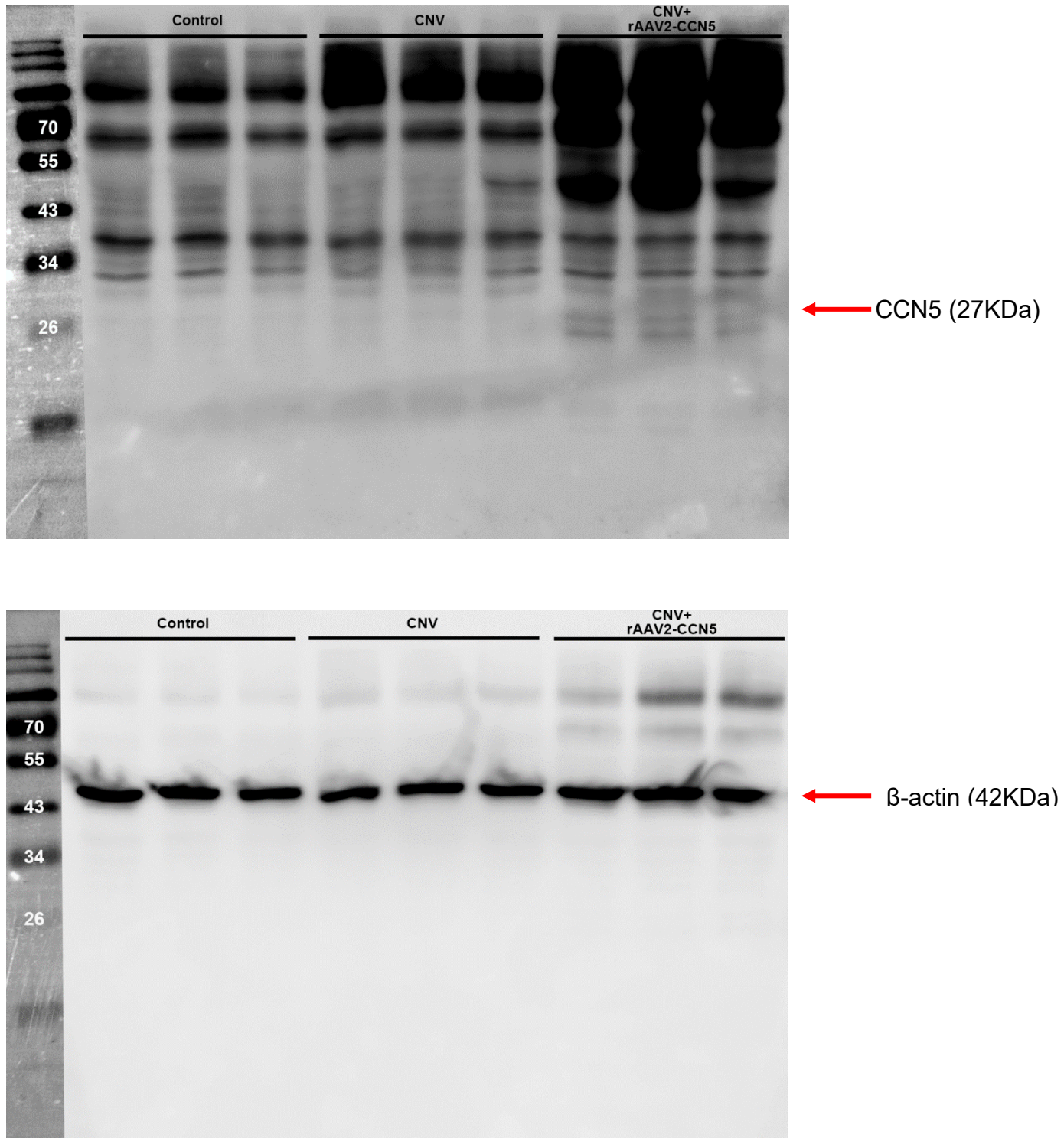

Figure 1-C

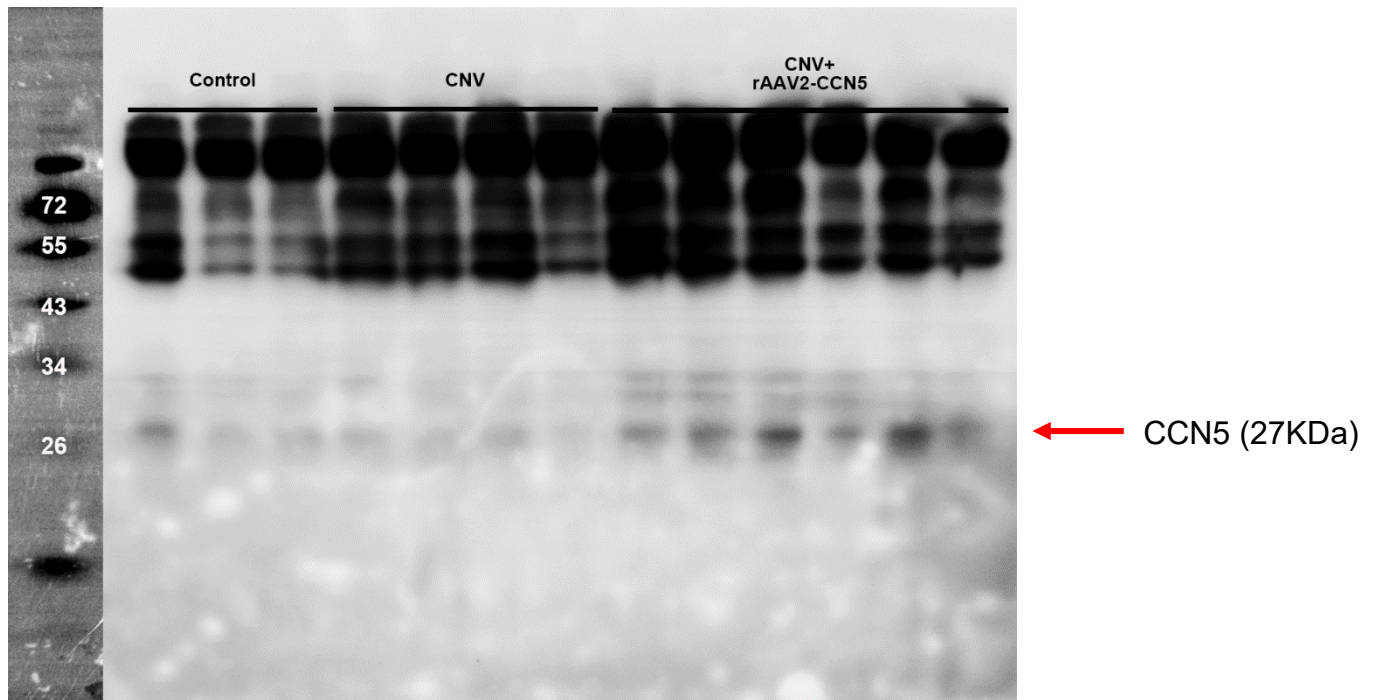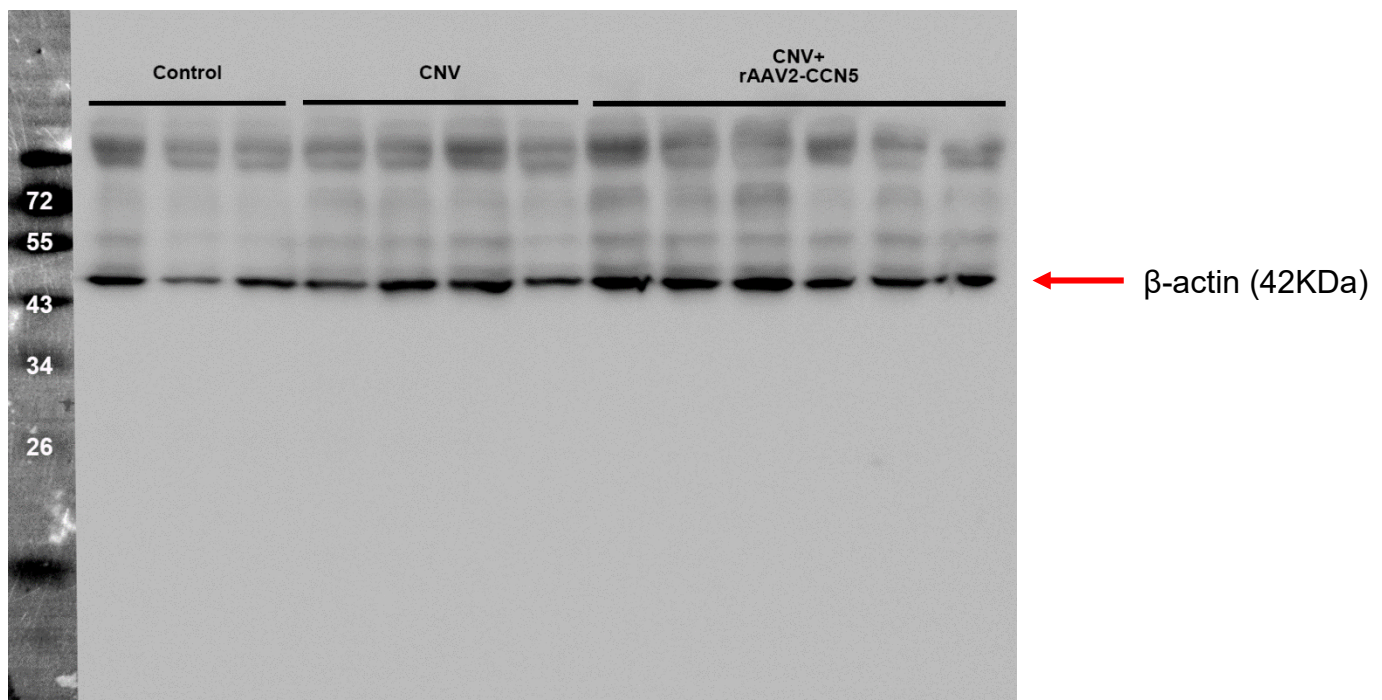

Supplementary Figure 1-C

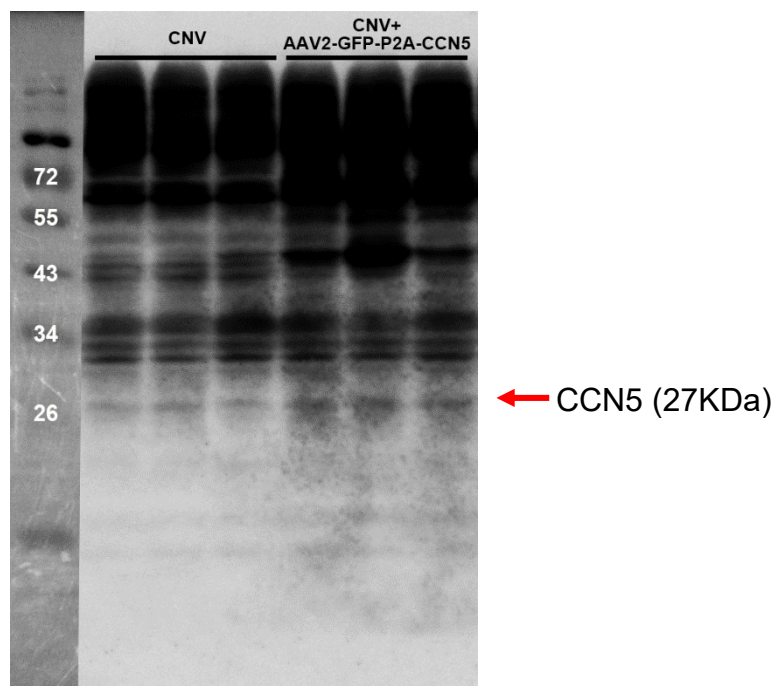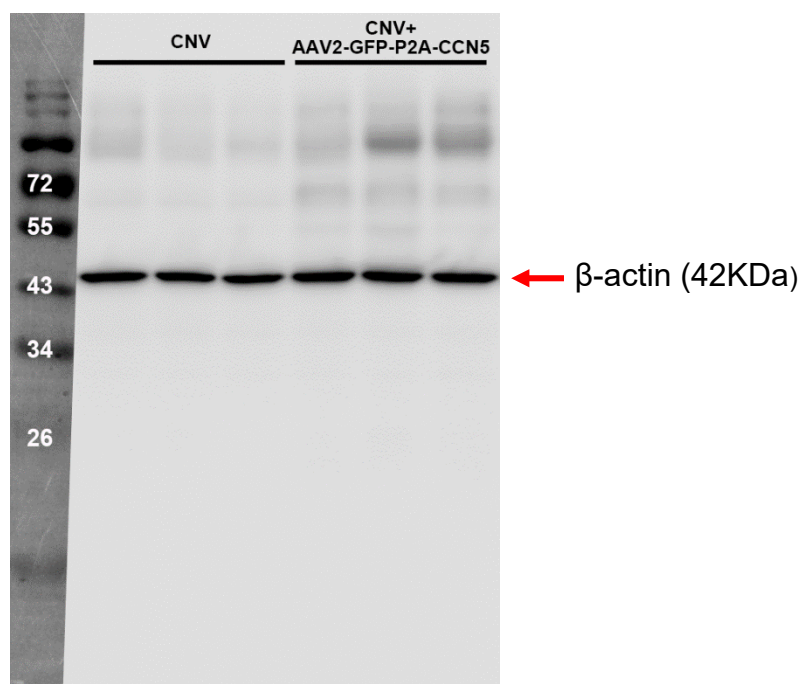

Supplementary Figure 1-D

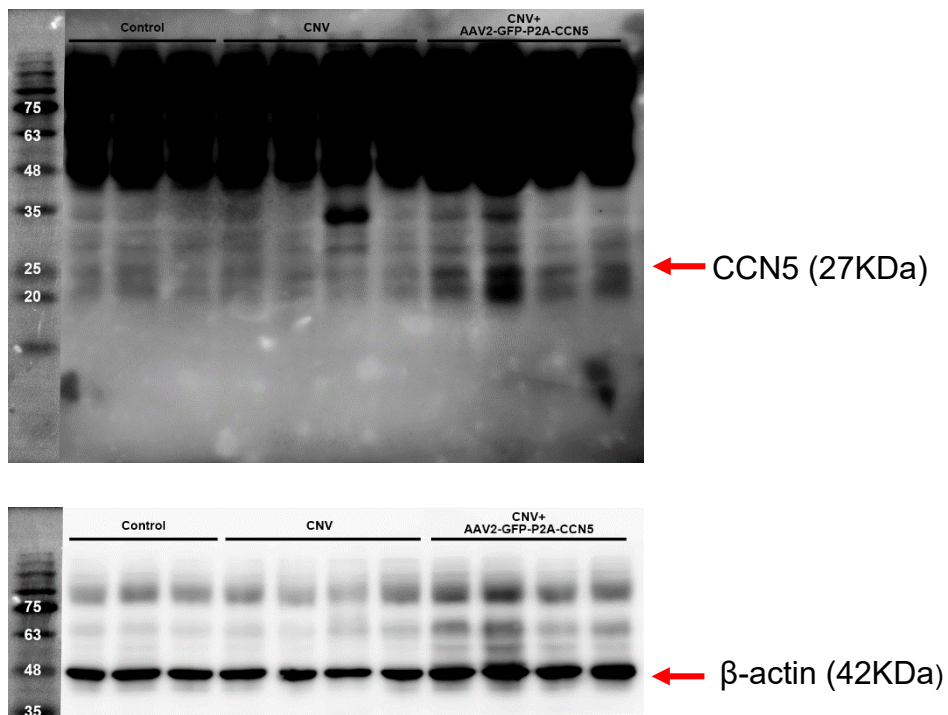

Supplementary Figure 2

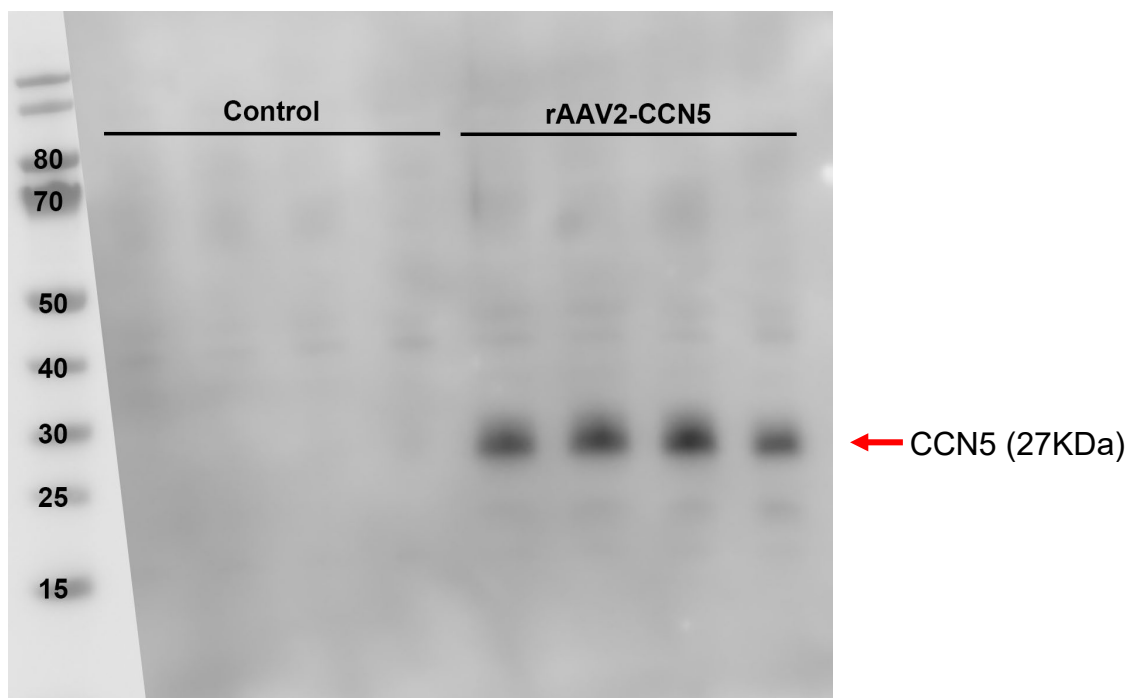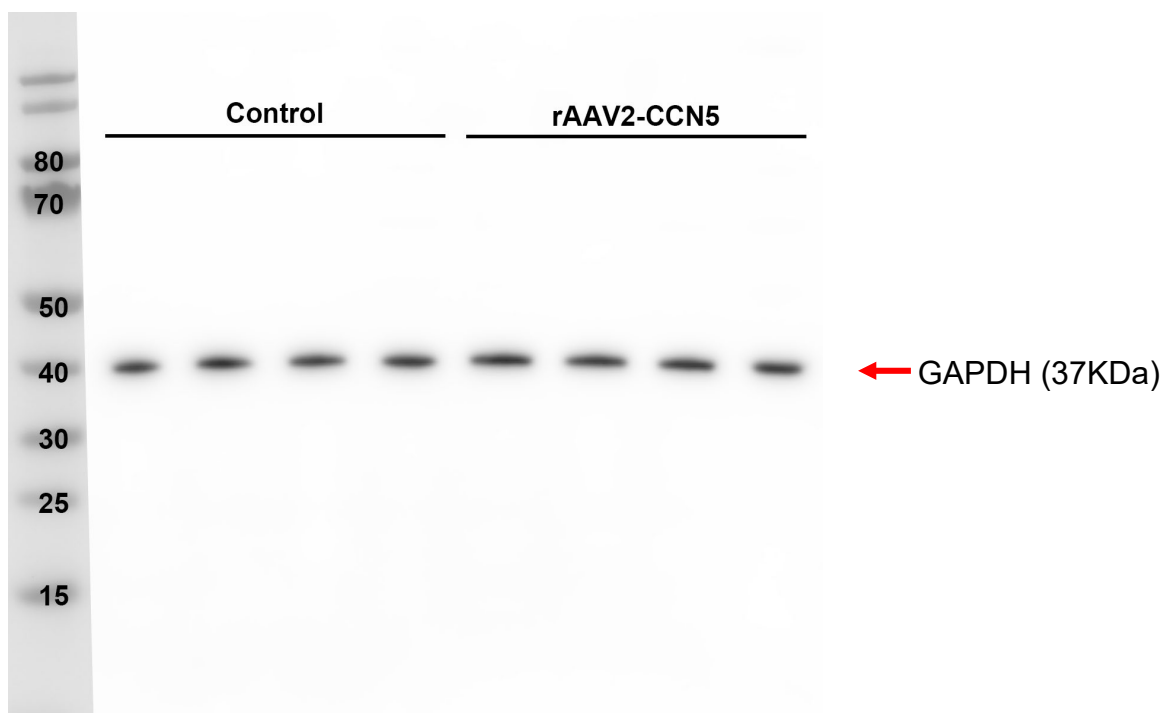

Supplement: S1 Raw images — (PDF) [file pone.0269937.s003.pdf]
